# Features of home and neighbourhood and the liveability of older South Africans

**DOI:** 10.1007/s10433-015-0343-2

**Published:** 2015-05-30

**Authors:** Suzan van der Pas, Serela Ramklass, Brian O’Leary, Sharon Anderson, Norah Keating, Bilkish Cassim

**Affiliations:** 1 Department of Epidemiology and Biostatistics, VU University Medical Center, Amsterdam, The Netherlands; 2 Department of Geriatrics, University of KwaZulu-Natal, Durban, South Africa; 3 eThekwini Municipality, Durban, South Africa; 4 Department of Human Ecology, University of Alberta, Edmonton, Canada; 5 Centre for Innovative Ageing, Swansea University, Swansea, UK; 6 Africa Unit for Transdisciplinary Health Research (AUTHeR), North-West University, Potchefstroom, South Africa

**Keywords:** Home, Liveability, Life satisfaction, Neighbourhood, Older adults, South Africa

## Abstract

While older people live in developing countries, little is known about the relative importance of features of their communities in influencing their liveability. We examine components of home and neighbourhood among older South Africans. Linear regression analyses revealed that features of home (basic amenities, household composition, financial status and safety) and neighbourhood (ability to shop for groceries, participate in organizations and feel safe from crime) are significantly associated with life satisfaction. Approaches to liveability that are person-centred and also set within contexts beyond home and neighbourhood are needed to address boundaries between home and neighbourhood; incorporate personal resources into liveability models and import broader environmental contexts such as health and social policy.

## Introduction

Population ageing and increasing concern about quality of life of older adults have drawn attention to the importance of place to older people. To a great extent, older adults conduct their daily lives close to home (Cho et al. 2012; Rowles and Bernard 2013; Wahl et al. 2012). There is considerable research from developed countries demonstrating the relevance to them of accessible homes, proximate services and features of the built environment including walkable neighbourhoods (Mahmood and Keating 2012; Wahl et al. 2012). Policy measures encouraging people to ‘age in place’ are increasingly prevalent (see, for example, Commonwealth Department of Health and Aging 2002; Farber et al. 2011) based on the assumption that remaining in one’s community enhances ageing well. Yet critics challenge this assumption noting that communities vary considerably in the resources, they have to support people as they age (Keating et al. 2013; Roos et al. 2014; Walsh et al. 2014).

The World Health Organisation’s initiative on age-friendly communities (2007) has moved this discussion of ageing and place to a global discourse with a call for enhancing knowledge of how communities around the world can provide environments that optimise quality of life of older adults. They draw attention to the rapid urbanisation of ageing and the need to better understand the immense challenges facing older people in the world’s cities (Plouffe and Kalache 2010). The majority of the world’s older urban residents live in developing countries often with poor living conditions, little public policy and minimal livelihood opportunities (Lloyd-Sherlock et al. 2012; Mberu et al. 2012). Yet despite these difficult conditions, there also is evidence that older persons may have a sense of attachment to their current residence (Falkingham et al. 2012). Given that increasing proportions of older people in Africa are urban dwellers, it seems important to determine elements of urban environments that might contribute positively to their quality of life. We draw on constructs of liveability to frame this question.

## Liveability

The term ‘liveability’ is used to describe the conditions that make communities good places to live (Hwang et al. 2008; Van Kamp et al. 2003; Veenhoven 2000). It was coined more than 50 years ago by North American and European environmentalists and urban activists worried that industrialization and rapid urban growth were destroying the quality of their neighbourhoods (Raad and Kenworthy 1998). Liveability reflected their aspirations to make the places where they lived more enjoyable and rewarding (Perkins 2005).

The concept of liveability has been adopted by researchers interested in people’s experiences of the quality of home and community environments. Liveability has been defined as ‘habitability’ of an environment (Veenhoven 2000); as the degree to which resources of the place meet the needs of residents and as satisfaction with the person–environment relationship (Biswas-Diener and Diener 2009; Van Kamp et al. 2003). Van Kamp et al. (2003) argue that liveability is an assessment by individuals of environmental features most relevant to their lives.

There is no consensus concerning the essential conditions of a liveable community (Kaal 2011) although specific elements may be more or less relevant to the constituents of interest. For example, economic forecasting firms (Mercer Client Services 2014; Economist Intelligence Unit 2012) have developed indices of urban liveability, such as climate or business conditions, used by employers to establish salaries or assign hardship allowances as part of job relocation. Urban planners focus on the natural and built environments as important criteria for good urban living (Vine et al. 2012a). Gerontologists consistently privilege home as the most important context for older persons (Gilleard et al. 2007) with increasing interest in neighbourhood as a supportive context.

The question then is how does one reconcile the implicit assumption that communities have features that make them liveable with the poor and deteriorating living conditions of some older urban dwellers in developing countries? Oswald et al. (2011) emphasise the relevance of this question when they argue that features of communities become more central to life satisfaction as increasing health restrictions with age necessitate remaining close to home. For older people in cities in sub-Saharan Africa, restrictions also may include the need to support proximate adult children and minimal opportunities to live elsewhere (Falkingham et al. 2012; Sharkey 2012). Yet we know little about components of liveability in such communities or which community features might contribute to life satisfaction. This study addresses the gap in understanding of liveability of older adults in developing regions by focusing on the components of liveability and their impact on life satisfaction among older South Africans in a city in KwaZulu-Natal (KZN), South Africa.

## Older adults in South Africa

South Africa has the highest proportion of older persons over the age of 60 years in sub-Saharan Africa, amounting to 3.9 million in 2011 (7.7 % of the population). The largest share (19 %) of older people resides in KZN (Statistics South Africa 2011). Households with older people in South Africa are among the most disadvantaged (Møller and Devey 2003). The African National Congress’s Reconstruction Development Programme (RDP) manifesto of 1994 focused on eradicating poverty through the investment in new houses, clean water and electricity, jobs and education and health opportunities. (ANC 1994). Although material standards of living are slowly improving for South African older households, many are still income poor and have less access to services such as piped water, electricity and sanitation compared to younger black households (Møller and Devey 2003).

## Components of liveability of older persons

There is a growing body of knowledge about elements of communities of older adults that are relevant to their lives. The focus is on home and on neighbourhood reflecting assumptions that lives of older people are lived close to home and that compared to younger people they suffer higher consequences from inadequate housing or lack of neighbourhood amenities (Haak et al. 2007; Wahl et al. 2012). Much of the research has been conducted in Europe, North America and Australia, where living conditions may differ considerably from those of older people in developing countries (Clayden et al. 2006; van Gent et al. 2009). In the next section of the paper, we review current knowledge of the components of home and neighbourhood that may influence older adults’ assessment of their liveability. Where possible we cite literature from sub-Saharan Africa.

### Home environment

In all world regions, home is seen as central to making an environment habitable (Veenhoven 2000). Researchers have found that, for older adults, home fosters identity and connectedness to those who age in familiar surroundings with meaningful possessions (Haak et al. 2007). Accessibility and convenience are features of home that are valued for their contribution to accomplishing daily activities (Cho et al. 2012). In contrast, research from developing countries, reviewed in the next section, is focused on basic household amenities, income adequacy and safety and on challenges to older people inherent in living in crowded multi-generation households. The research reflects the tenuous living situation of older persons in these regions.

#### Household amenities

Researchers in Southern Africa have found that basic household amenities such as indoor water and toilet, electricity and appliances affect older adults’ daily functioning and independence reducing their workload and increasing their capacity to cope with daily demands (Aigbavboa and Thwala 2012; Moolla et al. 2011; Ntema and Marais 2013). In recognition of housing needs of many residents, the South African Government has made a concerted effort to improve quality of housing. Homes built recently are more likely to be equipped with electricity, basic home appliances and indoor plumbing. The most common forms of access to clean piped water are direct access to the water on the site of the dwelling/yard and those that have access to a public tap off-site (Casale and Desmond 2007). Black African households generally have less access to piped water compared to households of White, Indian or Coloured South Africans^1^ (Casale and Desmond 2007).

#### Household composition

African policy discourses reflect a belief in the strength of families in supporting older adults (Hoffman et al. 2013). Cultural traditions and economic necessity in the region increase the likelihood of co-residence with adult children and grandchildren (Silverstein et al. 2012), although they reduce likelihood of living with a partner (Schatz et al. 2011). Households of older persons are large, multigenerational and predominantly female headed (Møller and Devey 2003). The main reasons for female headship are male labour migration and non-marriage (Posel 2001) but also premature death caused by HIV/AIDS (Gilbert et al. 2010). Multi-generation households of older persons are common since older adults play a key role in caring for grandchildren orphaned by HIV–AIDS (Ardington and Case 2010) or whose parents have migrated for work (Kahn 2011; Zimmer and Dayton 2005). Lack of privacy and daily disruptions in crowded households decrease older adults’ day-to-day coping and increase their dissatisfaction with housing (Baiden et al. 2011). At least one study has reported evidence of declining support to older adults from younger family members (Aboderin 2004).

#### Income

Prior studies in developed countries position income as a personal resource. Older adults with higher incomes have greater life satisfaction stemming from access to goods and services, leisure opportunities and a broad range of productive activities (Pinquart and Sorensen 2000). In contrast, for older South Africans, daily life for both themselves and others in their households can be constrained by lack of basic resources (Bohman et al. 2007). Nonetheless, South Africa is one of the few African nations to have a social pension scheme (Sagner 2000). South Africans aged 60 and above are eligible for a non-contributory pension funded from general government revenues (Schatz et al. 2014). Because of a women’s longer life expectancy, the pension reaches significantly more women than men (Burns et al. 2005).While almost 90 % of black older people in South Africa receive this pension, it often is pooled for poverty alleviation at a household level (Burns et al. 2005; Møller and Devey 2003; Schatz et al. 2012). In a context of high unemployment, some households of up to three generations exist on older adults’ old age pensions, straining generational relations (Klasen and Woolard 2009).

#### Safety in the home

Much of the European literature on home safety is about designing households to reduce risk of falls and other injuries (Iwarsson et al. 2009; Wahl et al. 2009). In contrast, research on home safety in developing countries most often relates to fear of violence. Older South Africans living in poverty experience substantial risk of exposure to physical and property violence in their homes (Ferreira and Lindgren 2008; Møller 2005). Financial abuse including extortion of pension and property theft is prevalent; over 90 % is perpetrated by a family member (Bohman et al. 2007; United Nations Population Fund (UNFPA) 2002). Paradoxically, home also is seen as a safe haven. In deprived areas, a strong front door creates perceptions of security (Bond et al. 2012).

In sum, research findings to date suggest that the home environment is as important to older people in South Africa as it is to older people in Europe. Yet as Bohman et al. (2007) note, for older South Africans, daily life can be a constant struggle suggesting that the most important components of home are those that contribute to safety, to lack of crowding and to basic comfort and convenience.

### Neighbourhood environment

In recent years, there also has been interest in features of neighbourhoods that are salient to older people. Oswald et al. (2011) argue that the built and social environments of neighbourhoods have an important influence on older adults’ full participation in their communities. Literature from developed countries suggests that neighbourhood features that are viewed as important to older adults fall into four broad domains: appropriate services (including shopping and health services), organizations (including seniors centres and churches), civility and safety (including trust and physical safety) and walkability (including distance to amenities and attractive surroundings) (Buys and Miller 2012; Julien et al. 2012; WHO 2007).

#### Appropriate services

Older adults are presumed to be more reliant than younger adults on local services and amenities because they are less mobile (Vine et al. 2012b). An element of housing policy in South Africa has been to locate services within local communities (Marais and Ntema 2013). Yet some communities have not benefitted from this strategy, and residents find few useful or valuable features of their neighbourhoods (Roos et al. 2014).

Even when local services are convenient, they may be neither adequate nor affordable. In a study of deprived areas in San Francisco, some older adults choose to bypass local shops and services, if they had transportation to get there, to shop at cheaper stores in suburban areas (Yen and Kaplan 2006). However, this is a resource generally unavailable to those living in poverty. Good transportation options for non-drivers are important in determining access to services (Buys and Miller 2012; Vine et al. 2012a, b). However, public transportation in South Africa is expensive, and there is limited penetration into communities (Cramm et al. 2012). Older people who live in neighbourhoods with higher incomes and socioeconomic status are more likely to experience greater overall life satisfaction (Biswas-Diener and Diener 2009; Yen et al. 2009).

#### Organizations

Participation in formal and informal organizations increases older adults’ social networks and social capital (Bohman et al. 2007; Cramm et al. 2012; Walsh et al. 2014). In Africa participation in stokvels (informal savings associations), religious organizations or burial/funeral societies are common among older adults (Bohman et al. 2007; Haddad and Maluccio 2003; Post and Mwangi 2009). The social elements and contacts made in these organizations are significant sources of financial, practical and emotional support for older adults. Due to the shortage of public support in South Africa, community activities are seen as important for older people who use them to create an informal support system (Bohman et al. 2007).

#### Civility and safety

Features of neighbourhoods such as perceptions of community safety and friendly neighbours have been shown to foster community cohesion and thus improve wellbeing (van Hooijdonk et al. 2007; Minkler 2010). Even in areas with high rates of poverty or crime, there are positive resources such as long-term friendships, knowledge of services and familiarity with the environment that promote quality of life (Minkler 2010; Pruchno et al. 2012).

Findings on the perceptions of neighbourhood trust and safety are mixed in Africa. Older residents in one low-income area were generally positive about mutual help among neighbours and neighbourhood safety, yet others were worried about crime (Post and Mwangi 2009). Loud music, graffiti, fights or exposure to hawkers and beggars increase older adults’ fears of crime and reduce satisfaction with the neighbourhood (Post and Mwangi 2009; UNFPA 2002). Alcohol and drug use among unemployed adults and youth are related to the incidence of crime and older adults’ fear of violence (Bohman et al. 2007; Ferreira and Lindgren 2008; Møller 2005).

#### Walkability

Being able to move safely about the community with easy access to shopping, health and community services are considered important elements of liveability. Australia has been a leader in walkability—creating neighbourhoods with opportunities for older adults to socialise as well as gain easy access to neighbourhood services. Researchers there have determined that walkable proximity for older adults is about 10 min walking distance from home, but this distance is affected by streetscapes including length of blocks, benches for resting and traffic density (Vine et al. 2012a). Yet environmental pressures in low-income neighbourhoods such as noise, crime, unemployment and lack of local amenities can decrease walkability for older adults (Buffel et al. 2013). The South African policy to increase tarring of roads and placement of community services (such as health centres and sports fields) within neighbourhoods can be seen as a recognition of their importance to the life satisfaction of residents (Ntema and Marais 2013).

In sum, research findings to date suggest that features of both home and neighbourhood may contribute to liveability among older people in deprived areas whose lives are lived close to home. Research suggests that the home environment is a particularly important component of liveability given discourses about the supportiveness of large multi-generation households in the face of evidence of deprived living conditions. The contribution of neighbourhood environment to liveability in developing countries is not well understood, though extant research suggests that neighbourhood safety and access to people and services are relevant to older adults.

## Methods

### Setting

The study was situated in the eThekwini Municipality located on the east coast of South Africa in the Province of KZN. The municipality spans an area of approximately 2297 km^2^ and is home to almost 3.5 million people. The townships of Inanda, Ntuzuma and KwaMashu (INK) are about twenty kilometres north of Durban City centre. The townships are adjacent to one another, have no physical boundaries and are managed through a single administrative unit of the eThekwini Municipality. The INK area is one of the oldest established townships and therefore likely to have a stable population.

INK is one of the most densely populated areas in South Africa with a population of approximately 580,000 persons (18 % of Durban’s population) living on 9340 hectares of land (South Africa Cities Network 2009). Of these, 6 % is over the age of 60 years. The area has high rates of poverty and unemployment, infrastructure is inadequate, and there is severe environmental degradation. About 40 % of the population are unemployed (The Business Trust 2007) and about 75 % of all households earn below South African Rand (ZAR) 9600 (€678) per annum; almost 25 % of incomes are below subsistence level. The area is well connected to the city by rail and highway, but travel within the area is limited and costly. The area is associated with high levels of violence and crime.

### Data source and sample

This study is a secondary analysis of data from a survey conducted to assess the determinants of health and quality of life in community dwelling older persons in the INK area. Data for the original study were collected by the University of KwaZulu-Natal (UKZN) in collaboration with the eThekwini Municipality Area-Based Management of the INK area and non-governmental organizations. Approval for the study was obtained from the Biomedical Research Ethics Committee of the University of KwaZulu-Natal, as well as from the Department of Health and local councillors following presentations at community forum meetings. At the meetings, the study was presented, and a focus group discussion was held with older members of the forum. Subsequently interviewers carried a letter stating that permission had been granted for the study to take place.

A two stage cluster sampling was used representing proportionate samples of older persons in the wards in each of the three areas (Inanda, Ntuzuma and KwaMashu) and of the proportions living in formal and informal housing. The criteria for inclusion in the sample were age 60 years and older, isiZulu or English speaking and the ability to consent. Systematic sampling was performed at street level. The starting points were defined using Geographic Information System co-ordinates in the three townships for both formal and informal settlements. Starting at a defined point, the field interviewer selected every fourth dwelling on the same side of the street to identify eligible respondents. The process continued on one street until a maximum of 25 % of the quota for the segment of the ward was obtained, before the field interviewer repeated the process on another street.

Face-to-face interviews were conducted by trained fieldworkers in either English or isiZulu as preferred by the respondent, after written consent was obtained. When the respondent was illiterate, a thumb print replaced written consent. Questionnaires were coded to ensure anonymity of the respondents. In each household, one South African citizen aged 60 years and older was recruited. A kish grid^2^ (Lewis-Beck et al. 2004) was used when more than one person was eligible. In total, 1008 older adults were recruited, and all agreed to participate in the study. The high response rate can be attributed to previous exposure to household surveys and the high levels of knowledge of the study resulting from the lengthy period of consultation with all stakeholders in the community. Variability in response rates from the different ethnic groups in South Africa has been reported (Shisana et al. 2013). In the SANHANES-1 study, the response rate in the African group was the highest at 88 % compared to 46 % in whites.

### Measures

#### Dependent variable

*Life satisfaction* is a conscious cognitive judgment of one’s life in which the criteria for judgment are up to the person (Diener et al. 1985). We used two life satisfaction questions from the original survey: (1) ‘taking everything into account are you satisfied with your life lately?’ and (2) ‘are you satisfied with the life you have led until now?’ The response categories ranged from (1) ‘very dissatisfied’ to (5) ‘very satisfied’. A sum of the two individual questions was made, ranging from 2 (very dissatisfied) to 10 (very satisfied) (Deeg 2007; Jonker et al. 2008, 2009).

#### Independent variables

*Home environment* was measured by the presence of household amenities, household composition, financial resources and safety. Household amenities were measured by a set of dichotomous variables concerning whether the respondent had access to and that they were in working order: electricity (no–yes), flush toilet inside (no–yes), water source (tap outside–tap inside), plus a count of appliances (stove, refrigerator, washer, radio, TV, telephone). Household composition was measured by assessing who was living in the household with the respondent. Based on the most predominant household types of black South African households (Amoateng et al. 2007), three post hoc categories were created: living alone, living without a partner but with children and other family member and living with a partner, children and other family members. Household size was measured by the number of people in the household. Financial resources encompassed 3 questions: total monthly household income, main source of income and evaluation of household finances compared to 2 years ago (worse, same or better). Monthly household income took into account all types of income per household, including social benefits, pensions and salaries. The responses ranged from no income to 25,600 ZAR (€1811). For the descriptive statistics, the responses were grouped into three categories: <1600 ZAR (<€116), 1601–3200 ZAR (€117–226) and >3201 ZAR (>€227). One question assessed feeling of safety at home; response categories ranged from (1) none of the time to (4) all of the time.

*Neighbourhood environment* was assessed by service availability, community engagement and safety. Service availability was measured by the availability of and access to health services and groceries. Availability of health care services included doctor (private, local hospital, local clinic), local clinic (nurse), ambulance service and community health worker. Mode of transport to health care facility included walking or private/public transport. Time to nearest health care facility (minutes) and ability to shop for groceries (no, yes) were used as proxies for accessibility to services in the community. Community engagement was assessed by the number of organizations that the respondent was involved in and frequency of participation (never, weekly to monthly). Questions about ability to walk outside for daily activities and feeling safe from crime in the neighbourhood evaluated neighbourhood safety.

#### Control variables

*Socio*-*demographic and health* variables (age, gender, level of education and self-rated health) were included as controls. Education ranged from no schooling to Standard 8 or higher qualification. Self-rated health was assessed by the question: ‘In general how would you say your health is at present?’ Response categories were (1) poor, (2) average, (3) good and (4) very good.

### Data analysis

Descriptive analyses were performed to describe the characteristics of the study sample. Multivariate regression models were used to examine the contribution to life satisfaction scores of two sets of explanatory variables: home environment and neighbourhood environment. Data were checked for missing observations. Because the missing data were minimal, complete data sets were used. Blocks of variables were entered into the models in the following order: (1) socio-demographic and health characteristics, (2) home environment and (3) neighbourhood environment. The final model included all variables from models 1 to 3. Statistical analyses were performed in IBM SPSS Statistics (version 20.0).

## Results

Descriptive statistics for the independent and dependent variables can be found in Table 1. The study population consisted of older adults with an average age of 68.9 years; over three quarters were women (77 %). Overall, respondents had relatively poor personal resources. The largest proportion (60 %) had three to 6 years of education, but one quarter (26 %) had no schooling at all. Forty percent (41 %) rated their health as good or very good. The mean score for life satisfaction was 5.4 (range 2–10).

**Table 1:** Characteristics of the study sample (n = 1008)

	%	Mean	SD	Range
*Socio-demographic and health characteristics*				
Age in years		68.9	7.37	60–103
Gender (female)	77.3			
Level of education		2.8	1.5	1–7
No schooling	26.0			
Up to Standard 7	59.0			
Standard 8 or higher	15.0			
Self-rated health		2.3	.9	1–4
*Home environment*				
Dwelling is ‘permanent home’	94.7			
Dwelling type				
Formal housing	53.8			
Informal housing	41.4			
Squatter housing	4.7			
Household amenities				
Electricity (yes)	95.0			
Number of appliances		4.2	1.4	0–6
Toilet (inside)	34.3			
Water (tap inside)	41.2			
Household composition				
Alone	9.6			
No partner but children/family	69.7			
Partner plus children/family	20.6			
Household size		3.5	1.5	0–11
Monthly household income				
0–1600 ZAR (0–116 EURO)	65.4			
1601–3200 ZAR (117–226 EURO)	24.8			
>3201 ZAR (>227 EURO)	9.8			
Main source of income				
Self	88.9			
Spouse	4.6			
Son/son-in-law	2.8			
Daughter/ in law	3.7			
Grandchild	.1			
Financial situation				
Worse	30.5			
Same	56.3			
Better	13.2			
Feel safe at home		3.2	.8	1–4
*Neighbourhood environment*				
Availability of health services		4.0	1.7	0–6
Time to health services (>31 minutes)	30.2			
Transport mode				
By foot (walking)	21.9			
Public/private transport	78.1			
Shopping for groceries (yes)	88.6			
Organizational involvement				
No involvement	26.7			
1+ organizations	73.3			
Meeting attendance				
Once a week or more	34.8			
Once a month or less	23.8			
Never	35.0			
Walking outside (yes)	86.0			
Feel safe from neighbourhood crime		3.2	.8	1–4
Life satisfaction		5.4	2.3	2–10

Respondents’ home environments also were challenging. Almost half lived in informal or squatter housing with no security of tenure and often no legal access to services such as water or electricity. Most (95 %) described their dwelling as their permanent home. Most reported having electricity in their homes (95 %) and having a number of electrical appliances that were functioning (Mean 4.2, SD = 1.4, range 0–6). A minority (34 %) had a toilet or water tap (41 %) inside the dwelling.

Overall, participants were not isolated. Few (10 %) lived alone. The majority (70 %) lived with adult children and other family members including grandchildren. The average household size was 3.5. However, the vast majority (89 %) provided the main source of income for their households. Incomes were modest. Two-thirds (65 %) specified their household monthly income from 0 to 1600 ZAR (0 to €116). Over half (56 %) rated their financial situation as the same as 2 years ago, just under a third believed their financial situation was worse (30 %) and 13 % thought they were better off. Feeling safe at home was relatively high (mean 3.2; range 1–4).

In terms of neighbourhood amenities, participants were aware on average of four health services (SD = 1.7), but for some, these were not accessible. Approximately, one-third (30 %) reported having to travel more than half an hour to their nearest health care service. Most (78 %) used some sort of public (bus, train, taxi) or private transport. The majority did their own grocery shopping (89 %).

Almost three quarters (73 %) of participants were involved in one or more organizations and over a third (35 %) attended meetings or activities of these organizations at least once a week. The majority were able to walk outside to do their daily activities (86 %). Feelings of safety from neighbourhood crime were reasonably high (mean 3.2; range 1–4, SD = .8).

### Home and neighbourhood characteristics and life satisfaction

Table 2 displays the standardised coefficients β for the multivariate regression analyses of life satisfaction. Three models were run. In model 1, socio-demographic and health characteristics were entered as control variables. In model 2, home environment characteristics were entered. In model 3, neighbourhood environment variables were added. Model 3 was the final full model with all blocks of variables.

**Table 2:** Association of life satisfaction with socio-demographic and health characteristics, home and neighbourhood environment: standardised coefficients (beta) and level of significance (*p* value) (n = 925)

	Model 1	Model 2	Model 3
*Socio-demographic and health characteristics*			
Age	.06	.05	.04
Female gender	−.02	−.02	−.04
Education	.13***	.10***	.07*
Self-rated health (poor–very good)	.49***	.32***	.25***
*Home environment*			
Household amenities			
Electricity		.06*	.08**
Appliances (0–6)		.04	.02
Toilet (inside–outside)		−.06*	−.04
Water (indoor tap–outdoor tap)		.16***	.14***
Household composition			
No partner, with children/family vs alone (ref)		.09	.10**
Partner, with children/family vs alone (ref)		.04	.05
Household size		.01	−.02
Financial resources			
Monthly household income		−.13***	−.10**
Income source (self–other)		.05	.05
Evaluation of finances (worse vs same/better)		.24***	.20***
Safety and security			
Feeling safe at home (none of the time vs all of the time)		.24***	.15***
*Neighbourhood environment*			
Service availability			
Availability of health services (no–yes)			−.05
Time to health services (minutes)			−.17***
Transport mode (on foot vs public/private transport)			.03
Ability to shop for groceries (no–yes)			.11***
Community engagement			
Involvement in organizations (none vs 1 or more)			.05
Participation rate in organizations (none vs weekly/monthly)			.10**
Walkability and safety			
Walking outside to shop (no–yes)			.02
Feeling safe from crime (none of the time vs all of the time)			.13**
R^2^ adj.	.26	.42	.47

* p < .05; ** p < .01; *** p < .001

In model 2, variables related to household amenities, financial resources and safety contributed to the explained variance. None of the household composition variables were significant.

Among household amenities, having electricity and an inside toilet were positively associated with life satisfaction. Yet having outdoor water was positively related to life satisfaction. Among financial resources, the belief that compared with 2 years ago the older adult’s financial status remained the same or improved was positively associated with life satisfaction. Yet having a higher total monthly income was negatively related to life satisfaction, a finding that seems at odds with evidence that financial capability provides access to resources. Reduced life satisfaction may result from normative pressure to share their pensions with household members and the risk of financial abuse when the older person has the only household income (Bohman et al. 2007).

Feeling safe at home also was positively correlated with life satisfaction. Yet none of the household composition variables were significantly associated with quality of life. Although families form an important part of daily routines, heavy financial and caring responsibilities, particularly for older women, may offset potentially positive experiences of being surrounded by kin. Variance explained in model 2 was 42 %.

In model 3, service availability, community engagement, safety and walkability contributed to the explained variance. Among service availability variables, the two significant associations with life satisfaction were amount of time to get to the nearest health care service and ability to shop for groceries without help. Both point to issues of accessibility. Presence of health services alone was not sufficient to serve as a resource that met the needs of participants, given the often lengthy travel time to reach health services. The ability to shop for groceries is particularly important for study participants who were responsible for the welfare of other people in their households.

Community engagement also was related to life satisfaction. Regular participation in organizations was significant, perhaps reflecting the longstanding tradition in South Africa of membership in organizations such as burial societies or stokvels (savings clubs) that are important in building and maintaining social capital in deprived neighbourhoods. Finally, feeling safe from crime was positively associated with life satisfaction.

In this final model, one household composition variable became significant. Those with no partner, but with adult children and other family members living in the household had a higher life satisfaction compared to those who are alone. Total explained variance of the full model was 47 %.

## Discussion

A question raised in this paper was how the assumption that communities have features that make them liveable can be reconciled with the deprived conditions of older urban dwellers. Insights from our findings suggest that, while we have begun to address this apparent paradox, there is need to further articulate and extend our conceptualization of liveability of older people.

### Home and neighbourhood liveability

Our main finding is that the near environments of home and neighbourhood are significantly associated with life satisfaction. Within the home environment, household amenities, people, financial resources and safety were all significantly associated with life satisfaction. Similar themes were found in the associations of features of the neighbourhood: services and amenities, people and safety.

These findings are consistent with gerontological research that positions both home and neighbourhood as important contexts for older persons. In accordance with Bohman et al. (2007) contention that daily life is difficult, our findings of important features of the near environment illustrate that older adults in our study find home and neighbourhood most liveable when these environments are safe and provide basic comfort, convenience and resources.

While consistent with previous research on the importance of home and neighbourhood, our findings also are somewhat at odds with assumptions from developed countries related to the important elements of home and neighbourhood. In that work, home often is positioned as a place in which people connect to memories, find comfort in familiar objects and recreate identity (Rowles and Chaudhury 2005). In turn, neighbourhoods are social places of public life (Gardner 2011) supported by age-friendly features of green spaces, lighting, benches, etc. (Vine et al. 2012b). In our study, there is little sense of positive connection to either home or neighbourhood where themes of liveability are more linked to safety and access to basic resources.

The finding that outdoor water access was positively associated with life satisfaction seems counterintuitive since convenience and safety afforded by having clean water in one’s home are viewed as constituting a major gain to the poor. However, at present, free water to dwellings is provided in eThekwini only to the poorest residents (Sutherland and Lewis 2012). For the majority, it is more likely that they now may have access to clean water in line with RDP standards of public taps within 200 metres from the dwelling (Møller and Devey 2003). For them, a reliable water supply may well be associated with life satisfaction. Also, we found that a higher total monthly income was negatively related to life satisfaction. This seems at odds with evidence that financial capability provides access to resources. Reduced life satisfaction may result from normative pressure to share their pensions with household members and the risk of financial abuse when the older person has the only household income (Bohman et al. 2007). Studies suggest that shifts take place in household types when older Black Africans become pension eligible (Schatz et al. 2014). The multigenerational household might be formed so that older persons can care for and provide income to extended kin (Schatz et al. 2014). These topics need further investigation including examining which factors impact the use of water in daily life as well as how older people make decisions about expenditures in multi-generation households.

Our findings may result in part from the historical context of apartheid that has left a mark on the township experience of the INK. Older people have lived through a period when their communities were among the least desirable of places. As Kaal (2011) would say, environments of the older people in this study have none of the criteria essential for a liveable community. Research in other settings in South Africa has shown that older adults living in such communities have little connection to place, and they see their neighbourhoods as being bereft of services and places that might benefit them (Roos et al. 2014).

Part of the reason for our findings also might lie in our interview protocol. In deference to the deprived living conditions of participants in this study, they were not asked about aesthetics of their settings, about community walkability, length of residence or connection to place. We need to think further about the meaningfulness of home and neighbourhood to people living in places not of their choosing. And we need to challenge the ‘received wisdom’ of home and neighbourhood as places of comfort and support.

Another important reason for our findings may come from the gendered experiences of ageing in South Africa. South African society predominantly positions women as vulnerable poor, with heavy responsibility for family caregiving, victims of violence, having little access to education or paid employment (Mosoetsa 2005). Gender was not significant in our multivariate models. However, given women’s and men’s considerable differences in family connections and household responsibilities, future research might explore the relative importance of aspects of home and community as well as broader contexts such as employment opportunities in liveability for older women and men.

### Liveability in context

In an earlier section of this paper, we cited Van Kamp et al. (2003) who define liveability as an assessment by individuals of environmental features most relevant to their lives. The gerontological tradition of documenting the importance of home and neighbourhood in the lives of older people and our findings of considerable proportions of variance explained by these two environments suggests that the Van Kamp and colleagues’ definition is apt. It is individuals themselves who are in the best position to assess the liveability of their communities.

Yet our findings also suggest that a definition of liveability based solely on a personal assessment of the most relevant environmental features is insufficient. Broader contexts are at play that constrain or increase the potential for near environments of neighbourhood and home to be experienced as liveable. Perhaps foremost among these is the historic context that led the INK to be designated as an area of severe environmental degradation. There is much to be learned as well about contemporary contexts. How do old age pension policies influence the prevalence and depth of poverty? How might better access to employment for younger adults reduce financial strain in households? Might barriers to inclusion in amenities of the broader community such as transportation be amenable to municipal policy interventions? The influence of many of these contexts is indirect and may be invisible to older adults whose near environments have a more obvious influence on their daily activities. As Phillipson (2007, p. 337) notes, “macro-sociological and economic forces, including globalisation, work on the ground to influence the daily lives of older people as well as the neighbourhoods in which they live”. It is at these more macro levels that policy interventions could make a difference in creating places that are more liveable to older adults.

In conclusion, we believe that our study has highlighted some of the challenges inherent in home and neighbourhood environments of older people. Themes of safety, access to basic amenities and to material resources are similar across home and neighbourhood environments. This suggests that the conceptual boundaries between them should be reconsidered. Approaches to liveability that are person-centred and yet set within contexts beyond home and neighbourhood may prove fruitful in setting research policy agendas that will have most impact on liveability of older people in deprived areas.

## Footnotes

1 The reference refers to a study comparing Black Africans with White, Indian and Coloured African households. These designations are used officially in South Africa to designate racial/ethnic origin. *Census 2011: Census in brief*. Pretoria: Statistics South Africa. 2012, https://www.statssa.gov.za/Census2011/Products/Census_2011_Census_in_brief.pdf. Retrieved 20 March 2015.

2 The Kish grid is a technique used in equal-probability sampling for selecting cases at random when more than one case is found to be eligible for inclusion when the interviewer calls at a sampled address or household.
